# Editorial: Cell cycle modulators: regulating the basic unit of life for disease treatment and tissue regeneration

**DOI:** 10.3389/fphar.2025.1554529

**Published:** 2025-01-29

**Authors:** Roberta Giordo, Audra N. Iness, Udhaya Kumar

**Affiliations:** ^1^ Department of Biomedical Sciences, Faculty of Medicine and Surgery, University of Sassari, Sassari, Italy; ^2^ Department of Medicine, Baylor College of Medicine, Houston, United States

**Keywords:** cancer, cell cycle regulation, multidisciplinary approaches, complex diseases, molecular mechanisms

Biomedical research has long focused on the complexity of cell cycle regulation and the molecular mechanisms underpinning various diseases. Recent advancements in this field have shed light on novel pathways, therapeutic interventions, and the interplay between genetic and environmental factors. This Research Topic brought together five studies to highlight the significance of multidisciplinary approaches for comprehending and addressing complex diseases, such as cancers, neurological disorders, and fibrotic conditions. These contributions not only enhance our understanding of cellular regulation but also open new avenues for targeted therapies.

In the context of cancer research, the study by Dindi et al. suggested that inhibition of sirtuin 6 (SIRT6) by imidazole derivatives may represent a novel promising therapeutic approach to circumvent Nrf2/Keap1-driven cellular rescue pathways in non-small cell lung cancer (NSCLC). SIRT6, which belongs to the sirtuin family of NAD + -dependent deacetylases, has emerged as an important player in encouraging cancer cells to survive under oxidative stress, confirming its role in redox-related cellular homeostasis. The authors demonstrate that imidazole derivatives trigger oxidative stress-mediated apoptosis in NSCLC cell lines through the Nrf2/Keap1 signaling pathway. This pathway, known for its role in cellular redox homeostasis, is modulated to induce apoptosis, thereby suppressing tumor progression. The findings underscore the potential of targeting SIRT6 as a therapeutic strategy, particularly in cancers resistant to conventional therapies. Future studies could explore the development of more selective inhibitors and their combinatory effects with existing treatments.

An interesting new therapeutic approaches, acting on the induction of ROS-dependent cell cycle arrest and oxeiptosis was suggested by Strusi et al. for the treatment of hepatocellular carcinoma (HCC), one of the most lethal forms of liver cancer. Precisely, authors investigated the synergistic effects of phenethyl isothiocyanate (PEITC) and dasatinib, two compounds with distinct mechanisms of action. PEITC, a natural compound derived from cruciferous vegetables, induces oxidative stress, while dasatinib, a tyrosine kinase inhibitor, disrupts cancer cell signaling. The combination of these agents results in enhanced cell cycle arrest and induction of oxieptosis, a form of regulated cell death driven by oxidative stress. This study not only highlights the potential of combinatory therapies in overcoming resistance but also advocates for the inclusion of natural compounds in therapeutic regimens. Future research could focus on optimizing dosage and delivery methods to maximize efficacy while minimizing adverse effects.

In their exploration of colorectal cancer (CRC), Aljuhani et al. focus on somatic mutations in BRAF, KRAS, and NRAS, key genes implicated in tumorigenesis. Using massively parallel sequencing, the study identifies actionable oncogenic variants and classifies them based on their therapeutic potential. These mutations appear to be not only CRC biomarkers but also important targets for therapeutic approaches in precision medicine. The authors emphasize the variation in mutation prevalence among populations, highlighting the need for focalized studies to develop customized treatments. By integrating genomic data with clinical outcomes, this research study bridges the gap between molecular biology and personalized medicine, offering a roadmap for future investigations into CRC-targeted therapies.

An abnormal arrest of cell cycle was observed by Wang et al. following chronic exposure to methamphetamine (METH) in hippocampal-derived neurospheres. In addition, METH caused abnormal differentiation, enhanced oxidative stress, and apoptosis in both neurons and astrocytes. These findings suggest that METH can disrupt the balance between proliferation and differentiation in neural progenitor cells, potentially leading to long-term cognitive and behavioral deficits. This study provides a framework for understanding METH-induced neurotoxicity’s cellular basis and highlights the importance of developing interventions to mitigate its impact on neurogenesis. Moreover, the employment of neurospheres as a model system highlights their utility in studying the effects of environmental toxins on neural development.

Fibrotic conditions such as keloids present significant challenges in clinical management due to their persistent and progressive nature. The work by Wang et al. provides a comprehensive review of the roles of matrix metalloproteinases (MMPs) 2 and 9 in the pathogenesis of keloids. These enzymes, involved in extracellular matrix (ECM) remodeling, are shown to interact with various signaling pathways to promote fibrosis. The authors discuss potential molecular targets exploitable in modulating MMP activity, including inhibitors that can prevent excessive scarring. This review not only consolidates existing knowledge but also identifies gaps that warrant further investigation, such as the interplay between MMPs and immune responses. This study’s implications extend beyond keloids, offering insights into other fibrotic diseases and potential therapeutic strategies.

Taken together, the studies featured in this Research Topic highlight the intricate interplay between molecular mechanisms and disease progression. From targeting specific oncogenic mutations to understanding the cellular impact of environmental toxins, these contributions underscore the importance of integrating basic and translational research. The emphasis on combination therapies, as demonstrated in the HCC study, reflects a growing recognition of the need for multi-pronged approaches in tackling complex diseases.

One of the recurring themes in these studies is the role of oxidative stress in disease pathology. Whether it is the induction of apoptosis in cancer cells or the disruption of neural progenitor cell function, oxidative stress emerges as a double-edged sword—a target for therapeutic intervention and a contributor to disease progression. Such an aspect highlights the need for a fine understanding of redox biology in the context of health and disease.

Translating these findings into clinical practice is another critical aspect that needs further exploration. In fact, while the studies provide compelling preclinical evidence, challenges remain in filling the gap between laboratory research and patient care. Future research should prioritize clinical trials to validate the efficacy and safety of the proposed interventions. Additionally, the precision and effectiveness of these therapies could be enhanced by the development of biomarkers to monitor treatment response.

The importance of methodological diversity in biomedical research is highlighted by the inclusion of diverse model systems, including cell lines and neurospheres. These models not only facilitate the study of disease mechanisms but also enable the testing of novel interventions in a controlled environment. However, complementing these studies with *in vivo* models and clinical data is crucial to ensure their relevance to human health.

By examining these advancements in the larger context of cellular and molecular biology, this editorial aims to promote innovation and collaboration in the scientific community. The Research Topic’s findings not only help us better understand cell cycle dynamics and molecular interventions, but also open the door for more effective and personalized treatments. Integrating multidisciplinary approaches will be crucial in addressing the complexities of human diseases and improving patient outcomes as we move forward.

